# Subtyping preserved ratio impaired spirometry (PRISm) by using quantitative HRCT imaging characteristics

**DOI:** 10.1186/s12931-022-02113-7

**Published:** 2022-11-11

**Authors:** Jinjuan Lu, Haiyan Ge, Lin Qi, Shaojie Zhang, Yuling Yang, Xuemei Huang, Ming Li

**Affiliations:** 1 Department of Radiology, Huadong Hospital Affiliated to Fudan University, 221 West Yanan Road, Jingan District, Shanghai, 200040 China; 2 Department of Respiratory Medicine, Huadong Hospital Affiliated to Fudan University, Shanghai, China

**Keywords:** Chronic obstructive pulmonary disease, Preserved ratio impaired spirometry, Pulmonary function test, Quantitative, Computed tomography

## Abstract

**Background:**

Preserved Ratio Impaired Spirometry (PRISm) is defined as FEV1/FVC ≥ 70% and FEV1 < 80%pred by pulmonary function test (PFT). It has highly prevalence and is associated with increased respiratory symptoms, systemic inflammation, and mortality. However, there are few radiological studies related to PRISm. The purpose of this study was to investigate the quantitative high-resolution computed tomography (HRCT) characteristics of PRISm and to evaluate the correlation between quantitative HRCT parameters and pulmonary function parameters, with the goal of establishing a nomogram model for predicting PRISm based on quantitative HRCT.

**Methods:**

A prospective and continuous study was performed in 488 respiratory outpatients from February 2020 to February 2021. All patients underwent both deep inspiratory and expiratory CT examinations, and received pulmonary function test (PFT) within 1 month. According to the exclusion criteria and Global Initiative for Chronic Obstructive Lung Disease (GOLD) classification standard, 94 cases of normal pulmonary function, 51 cases of PRISm and 48 cases of mild to moderate chronic obstructive lung disease (COPD) were included in the study. The lung parenchyma, parametric response mapping (PRM), airway and vessel parameters were measured by automatic segmentation software (Aview). One-way analysis of variance (ANOVA) was used to compare the differences in clinical features, pulmonary function parameters and quantitative CT parameters. Spearman rank correlation analysis was used to evaluate the correlation between CT quantitative index and pulmonary function parameters. The predictors were obtained by binary logistics regression analysis respectively in normal and PRISm as well as PRISm and mild to moderate COPD, and the nomogram model was established.

**Results:**

There were significant differences in pulmonary function parameters among the three groups (P < 0.001). The differences in pulmonary parenchyma parameters such as emphysema index (EI), pixel indices-1 (PI-1) and PI-15 were mainly between mild to moderate COPD and the other two groups. The differences of airway parameters and pulmonary vascular parameters were mainly between normal and the other two groups, but were not found between PRISm and mild to moderate COPD. Especially there were significant differences in mean lung density (MLD) and the percent of normal in PRM (PRM^Normal^) among the three groups. Most of the pulmonary quantitative CT parameters had mild to moderate correlation with pulmonary function parameters. The predictors of the nomogram model using binary logistics regression analysis to distinguish normal from PRISm were smoking, MLD, the percent of functional small airways disease (fSAD) in PRM (PRM^fSAD^) and Lumen area. It had a good goodness of fit (χ^2^ = 0.31, P < 0.001) with the area under curve (AUC) value of 0.786. The predictor of distinguishing PRISm from mild to moderate COPD were PRM^Emph^ (P < 0.001, AUC = 0.852).

**Conclusions:**

PRISm was significantly different from subjects with normal pulmonary function in small airway and vessel lesions, which was more inclined to mild to moderate COPD, but there was no increase in pulmonary parenchymal attenuation. The nomogram based on quantitative HRCT parameters has good predictive value and provide more objective evidence for the early screening of PRISm.

## Introduction

Chronic obstructive pulmonary disease (COPD) is a chronic respiratory disease characterized by persistent respiratory symptoms and airflow restriction. It is known that its common pathogenesis is related to airway or alveolar abnormalities caused by frequent exposure to harmful gases or suspended particles in the air. Harmful external stimulation can cause irreversible airway stenosis and airway wall inflammation. The airflow limitation is the result of a prolonged time constant for lung emptying, caused by increased resistance of the small conducting airways and increased compliance of the lung [1]. These pathological processes are characteristic anatomic changes that are present in COPD and are functionally reflected as irreversible airflow restriction and decreased ventilatory function. Recent evidence from Hogg’s group shows that the pathogenesis of COPD is complex and heterogeneous and several mechanisms coexist and interact [2]. However, it is a common, predictable and treatable disease. The latest data show that the total number of COPD patients has reached 1/14 of the population in China [3], and COPD has become one of the three major causes of death in the world [4].

At present, the diagnosis of COPD is mainly based on the pulmonary function test (PFT), and its diagnostic standard is FEV1/FVC < 0.70 after inhaling bronchodilators. However, only when the lung tissue damage exceeds 30% and small airway obstruction exceeds 75%, will the PFT examination be abnormal [5]. The early diagnosis of COPD is still a clinical challenge because its clinical manifestations have individual differences. Some patients do not have any respiratory symptoms during the early stage, and there are obvious deficiencies in early diagnosis and standardized treatment with PFT. Studies [6] have shown that the earlier COPD patients receive treatment, the greater the recovery of pulmonary function, highlighting the importance of an early diagnosis.

One subtype of COPD is called preserved ratio impaired spirometry (PRISm), which is characterized by FEV1 < 80%pred and FEV1/FVC ≥ 70%, and this subtype is classified as pulmonary dysfunction that does not meet the spirometry definition of COPD. Therefore, such patients are largely excluded from clinical trials [7]. The detection rate of PRISm was 12.3% in a study of COPDGene [8]. Compared with patients with a normal lung function test, patients with PRISm had worse dyspnea, shorter walking distance, an increased risk for emphysema, decreased total vital capacity, and an increased segmental bronchial wall area percentage, and these patients were associated with increased respiratory symptoms, systemic inflammation and mortality. Another COPDGene study [9] found that patients with PRISm worsened more frequently and severely than mild COPD patients. However, there are few quantitative CT studies related to the early diagnosis of PRISm patients.

Based on deep inspiration and expiration HDCT chest images, this study performed a quantitative analysis of the lung parenchyma, bronchi, and pulmonary blood vessels in subjects with PRISm, with mild to moderate COPD, and with normal lung functional tests to explore the imaging features of PRISm and the early diagnostic value of quantitative HDCT.

## Methods

### Ethical considerations

This study was approved by the Ethics Committee of Huadong Hospital affiliated to Fudan University (Approve No. 2021K018). All patients have written informed consent.

### Patients

**Fig. 1:**
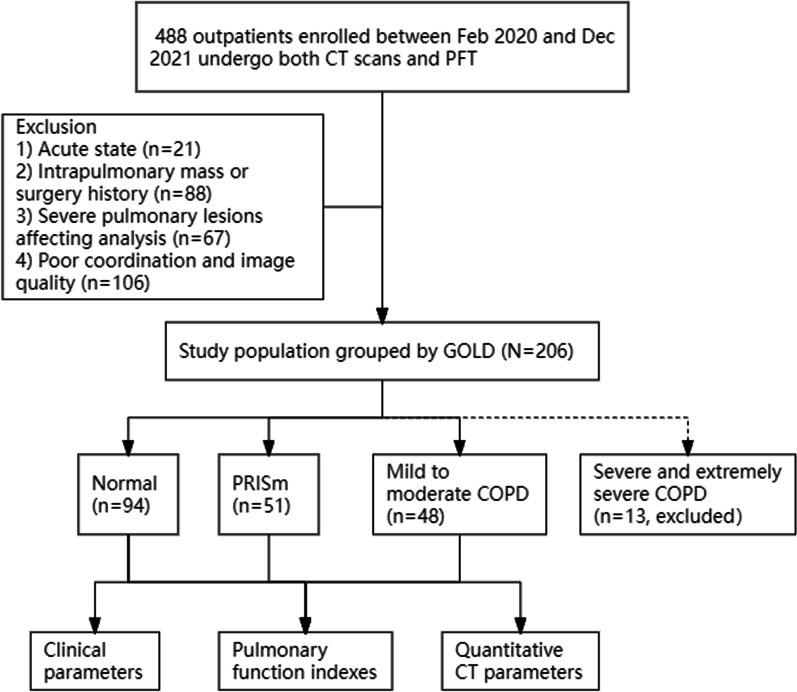
Flow diagram. *PFT* pulmonary function test, *GOLD* Global Initiative for Chronic Obstructive Lung Disease

This prospective study included 488 respiratory clinic patients from HuaDong Hospital affiliated with Fudan University from February 2020 to February 2021. The patients underwent both inspiration and expiration CT scanning and PFTs. The exclusion criteria were as follows: (1) acute attack of respiratory system (n = 21); (2) intrapulmonary mass or history of thoracic surgery (n = 88); (3) severe thoracic deformity, consolidation of lung, atelectasis, massive pleural effusion or severe pulmonary interstitial fibrosis affecting lung analysis (n = 67); (4) poor respiratory coordination and poor CT scanning image quality (n = 106). According to the 2021 Global Initiative for Chronic Obstructive Lung Disease (GOLD), the patients who finally met the requirements of the analysis were divided into normal group (n = 94), PRISm patients (n = 51), mild to moderate COPD patients (n = 48) (including GOLD grade 1 and 2), severe and extremely severe patients (including GOLD grade 3 and 4). Severe and extremely severe patients were excluded because the number of patients was small and there was a large clinical difference when comparing the severe patients to PRISm patients (Fig. 1). The grading standard of lung function is: normal, FEV1/FVC ≥ 70% and FEV1 ≥ 80% pred; PRISm, FEV1/FVC ≥ 70% and FEV1 < 80%pred; GOLD 1 (mild), FEV1/FVC < 70% and FEV1% ≥ 80% pred; GOLD 2 (moderate), FEV1/FVC < 70% and FEV1% ≥ 50% pred, < 80% pred; GOLD 3 (serve), FEV1/FVC < 70% and FEV1% ≥ 30% pred, < 50% pred; GOLD 4 (extremely severe), FEV1/FVC < 70% and FEV1% < 30% pred.

### Pulmonary function test and clinical parameters

The subjects underwent PFT (MasterScreen, Jaeger, Germany) within 1 month before or after CT examination. According to the guidelines of 2019 American Thoracic Society and European Respiratory Society [10], forced expiratory volume in one second (FVE1), percentage predicted forced expiratory volume in one second (FEV1%pred), forced vital capacity (FVC), ratio of forced expiratory volume in one second to forced vital capacity (FEV1/FVC), vital capacity (VC), carbon monoxide diffusion capacity (DLCO) and ratio of residual volume to total lung capacity (RV/TLC) were measured after inhalation of bronchodilators. Up to three measurements were made, and we took the best value. The measured values of VC and DLCO are expressed as a percentage of the measured value to the predicted value. In addition to the PFT indices, this study also evaluated basic parameters such as age, smoking, and body mass index (BMI).

### CT techniques and quantitative parameters

The 64-detector CT system (GE Discovery CT750 HD or Somatom Definition Flash) was used at full inspiration and expiration for all patients. They were informed of the purpose and method before CT scanning, and were given multiple respiratory training. The scanning parameters was recommended by the Fleischner Society [11]: Pitch: 1–1.4; acquisition collimation: ≤ 1 mm; Kilovolt peak:120; effective milliampere second: 40–200; reconstruction algorithms: smooth and sharp; reconstruction section thickness: 0.625–1 mm; reconstruction interval: 0.5–0.9 mm; reconstruction field of view: lungs only. The images were analyzed using automatic segmentation software (Aview, Korea). Emphysema index (EI) is the percentage of the low-density area in the deep inspiration phase to the total lung volume, with a threshold of − 950 HU. Air trapping index (ATI) and parametric response mapping (PRM) are obtained by registration of inspiratory and expiratory CT images. Pixel histogram is used to measure the mean lung density (MLD) in inspiratory phase and the pixel index (PI) when it accounts for 1% and 15% of the whole lung. In terms of airway, the square root of wall area of hypothetical airway with internal perimeter of 10 mm (pi10) and the wall thickness, wall area percentage and lumen area of Sixth grade bronchus were measured. In terms of vessels, we measured the total pulmonary vessel volume and some vascular parameters 6 mm away from the pleural surface, and the parameters included the number of vessels, the number of vessels under 5 mm^2^, the average diameter of vessels, the area of the vessels, and surface low attenuation area (LAA). All quantitative parameters were measured automatically by the software and were supervised by radiographers with more than 2 years of image processing experience.

### Statistical analysis

All statistical analyses were performed using a commercially available software program (SPSS 23.0 for Windows; SPSS, Chicago, IL, USA). A chi square test was used to analyze the categorical variables. Data pertaining to continuous variables are expressed as a mean ± standard deviation. Multigroup comparisons were performed with one-way analysis of variance (ANOVA) and a least significance difference (LSD) was used to compare between the two groups. Tamhane’s T2 nonparametric test was used when the variance was uneven. Spearman rank correlation analysis was used to evaluate the correlation between CT quantitative indexes and pulmonary function parameters. Binary logistic regression analysis of stepwise selection was carried out in normal with PRISm group, as well as PRISm with mild to moderate COPD group to determine the predictive factors. Use Medcalc (version 19.0.2) to draw receiver operating characteristic (ROC) curve and calculate the efficiency of model and predictors. The differences of ROC curves were compared by Delong test. R statistical software (version 3.5.1; http://www.Rproject.org) was used to draw nomogram and calibration curve. P values < 0.05 indicated statistical significance.

## Results

### Clinical and quantitative CT parameters

Table 1 summarizes the clinical data, PFT results and lung quantitative parameters of all of the patients. A total of 193 patients were evaluated, including the normal group (n = 94), PRISm group (n = 51) and mild to moderate COPD group (n = 48). There were significant differences in age, smoking status, pulmonary function parameters, pulmonary parenchyma attenuation parameters and some airway and vascular parameters among the three groups.

**Table 1:** Comparison of demographic, PFT characteristics and CT parameter among the three groups (n = 193)

	Normal n = 94	PRISm n = 51	Mild to moderate COPD n = 48	P value
Sex (male/female), n	58/36	30/21	36/12	0.189
Age (years)	62.3 ± 10.2	65.3 ± 12.1	68.0 ± 8.5**	0.008
BMI (kg/m^2^)	23.6 ± 3.2	23.6 ± 3.1	23.4 ± 3.3	0.924
Smoker (n, %)	32 (34.0%)	34 (66.7%)***	33 (68.8%)***	< 0.001
Pulmonary function parameters				
FEV1 (L)	2.6 ± 0.5	1.6 ± 0.4***	1.5 ± 0.3***	< 0.001
FEV1% predicted (%)	110.3 ± 19.6	70.5 ± 9.8***	66.5 ± 12.9***	< 0.001
FVC (L)	2.8 ± 0.6	1.9 ± 0.5***	2.5 ± 0.6**^###^	< 0.001
FEV1/FVC (%)	90.1 ± 6.7	85.4 ± 10.5^*^	60.0 ± 6.9***^###^	< 0.001
VC% predicted (%)	99.9 ± 14.8	75.5 ± 13.8***	86.4 ± 17.2***^###^	< 0.001
DLco% predicted (%)	85.7 ± 19.4	66.3 ± 19.1***	66.8 ± 26.0***	< 0.001
RV/TLC (%)	46.7 ± 7.7	53.1 ± 8.8***	56.3 ± 6.1***^#^	< 0.001
Parenchymal parameters on CT				
EI (%)	6.0 ± 4.7	6.6 ± 7.8	19.5 ± 11.8***^###^	< 0.001
ATI (%)	31.1 ± 24.1	41.7 ± 27.6	49.1 ± 25.3***	< 0.001
Inspiratory MLD (HU)	− 841.7 ± 29.2	− 823.8 ± 37.8**	− 857.8 ± 38.3**^###^	< 0.001
PI-1 (HU)	− 979.1 ± 20.6	− 976.6 ± 29.2	− 1006.5 ± 19.3***^###^	< 0.001
PI-15 (HU)	− 919.9 ± 22.0	− 912.5 ± 36.2	− 958.0 ± 33.0***^###^	< 0.001
PRM^Emph^ (%)	3.5 ± 3.4	4.5 ± 6.1	16.7 ± 11.4***^###^	< 0.001
PRM^fSAD^ (%)	15.4 ± 10.3	21.7 ± 14.2**	29.9 ± 19.2***	< 0.001
PRM^Normal^ (%)	81.1 ± 12.9	73.8 ± 18.4*	53.4 ± 24.2***^###^	< 0.001
Airway parameters on CT				
Pi10 (mm)	3.1 ± 0.7	3.6 ± 1.0**	3.6 ± 1.0**	< 0.001
Wall thickness (mm)	0.98 ± 0.39	1.01 ± 0.28	1.12 ± 0.31*	0.065
Wall area (%)	54.8 ± 10.2	60.4 ± 10.0**	63.2 ± 9.1***	< 0.001
Lumen area (mm^2^)	11.3 ± 5.0	9.2 ± 4.2*	9.3 ± 4.6*	0.009
Vessel parameters on CT				
Total vessel volume (cc)	219.0 ± 76.6	211.7 ± 123.7	239.6 ± 104.3	0.336
Number of vessels (ea)	1592.4 ± 680.2	1308.6 ± 697.6*	1270.2 ± 750.1*	0.012
Number of vessels under 5 mm^2^ (ea)	1437.4 ± 649.6	1138.8 ± 639.6*	1086.5 ± 721.8**	0.004
Average diameter of vessels (mm)	1.62 ± 0.32	1.75 ± 0.38*	1.81 ± 0.39**	0.006
Area of vessels (mm^2^)	4174 ± 1854	3822 ± 1916	4089 ± 2016	0.568
Surface LAA (mm^2^)	13,377 ± 12,152	15,252 ± 21,489	51,750 ± 36,007***^###^	< 0.001

Values were obtained using inspiration and expiration CT images

BMI: body mass index; PRISm: preserved ratio and impaired spirometry; COPD: chronic obstructive pulmonary disease; FEV1: forced expiratory volume in one second; FEV1% predicted: percentage predicted forced expiratory volume in one second; FVC: forced vital capacity; VC: vital capacity; DLCO: percentage of predicted diffusing capacity of the lungs for carbon monoxide; RV: residual volume; TLC: total lung capacity; EI: emphysema index; ATI: air trapping index; MLD: mean lung density; HU: Hounsfield unit; PI: pixel indices; Pi10: square root of wall area of hypothetical airway with internal perimeter of 10 mm; PRM: parametric response mapping; PRM^Emph^: the percent of emphysema in PRM; PRM^fSAD^: the percent of fSAD in PRM; PRM^Normal^: the percent of normal in PRM; fSAD: functional small airway disease

*P < 0.05, **P < 0.01, ***P < 0.001, compared to the group of normal; ^#^P < 0.05, ^##^P < 0.01, ^###^P < 0.001, compared to the group of PRISm

**Fig. 2:**
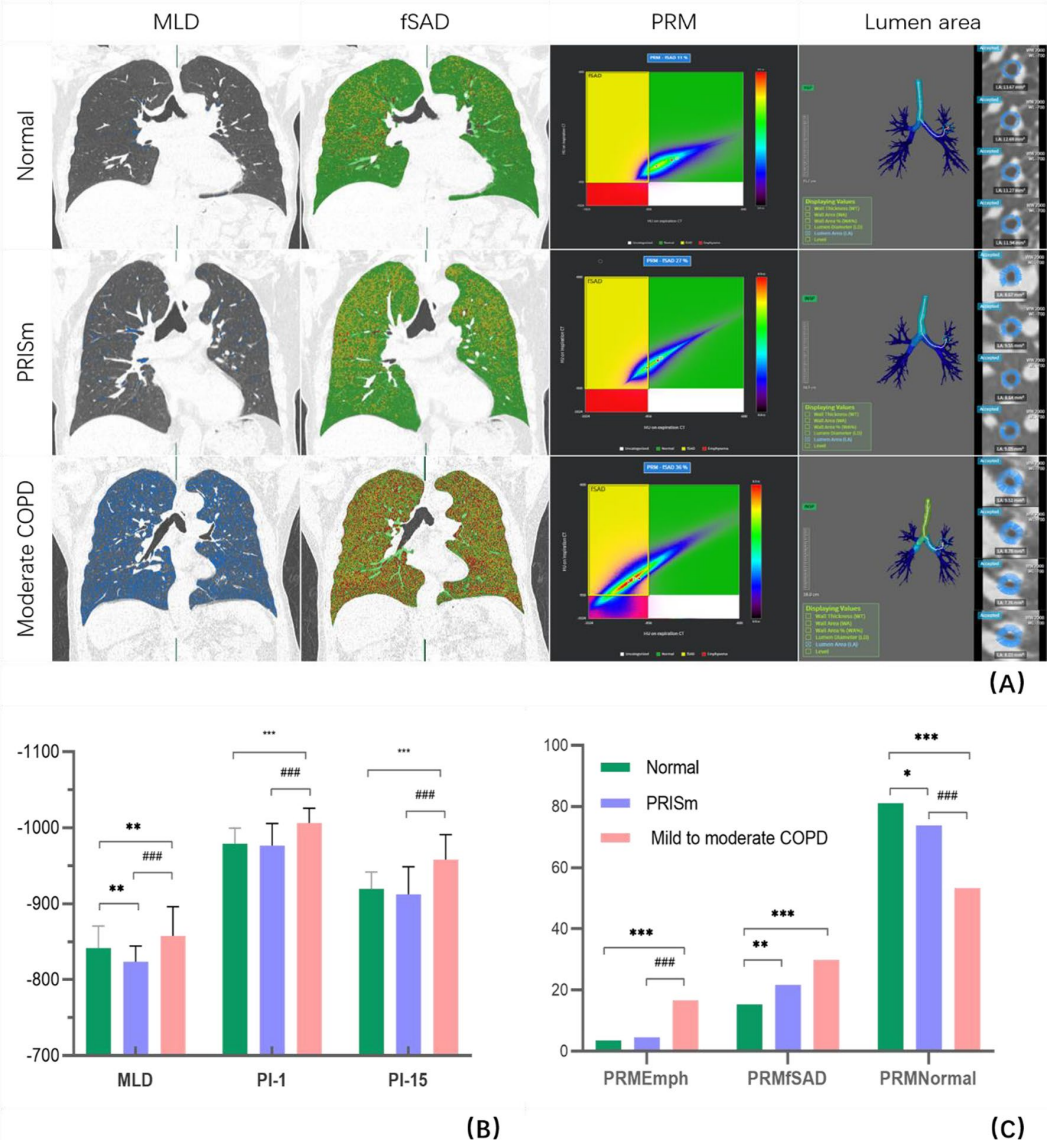
**A** The imaging measurement parameters for MLD, PRM^fSAD^ and lumen area of sixth bronchus in three patients with normal lung function, PRISm and moderate COPD. The blue color in the first column represents the area with a CT value less than − 950 HU on inspiratory CT. In the PRM obtained from inspiration and expiration CT, red represents the emphysema area, yellow represents the functional small airway disease area, and green represents the normal lung tissue area. The last column is the 3D bronchial tree, and the four small images on the right represent sections of the sixth bronchus. The normal patient was a 56-year-old man (FEV1%pred = 121.0%, FEV1/FVC% = 94.27%). The MLD was − 851.7 HU; the emphysema, fSAD and normal percent of PRM was 1.5%, 11% and 87.5%; the lumen area of the sixth bronchus was 10.4 mm^2^. The patient with PRISm was a 52-year-old man (FEV1%pred = 76.4%, FEV1/FVC% = 70.63%). The MLD was − 798.0 HU; The emphysema、fSAD and normal percent of PRM was 12%, 24% and 64%; The lumen area of was the sixth bronchus 5.4 mm^2^. The patient with GOLD 2 was a 68-year-old man (FEV1% pred = 58%, FEV1/FVC% = 69.11%). The MLD was − 863.6 HU; The emphysema, fSAD and normal percent of PRM was 29%, 36% and 35%; the lumen area of the sixth bronchus was 5.8 mm^2^. **B** The bar chart shows that there were significant differences in the parameters of MLD, PI-1 and PI-15 among the three groups of normal, PRISm and mild to moderate COPD, especially in MLD. **C** The bar chart shows that there were significant differences in PRM^Emph^ between PRISm and mild to moderate COPD, as well as PRM^fSAD^ between the normal and PRISm patients

However, there were no significant differences in sex, BMI, wall thickness when evaluating the airway parameters, the total vessel volume or the area of the vessels when evaluating the vessel parameters (P > 0.05). Through a pairwise comparison between the groups, we further found that there was no significant difference in the pulmonary function parameters such as FEV1, FEV1%pred and DLCO% between the PRISm group and the mild to moderate COPD group. There were significant differences in RV/TLC among the three groups, and the difference between PRISm group and normal group was more significant than that between PRISm group and mild to moderate COPD group. The parameters of the pulmonary parenchyma, such as ATI and functional small airway disease (fSAD), which reflect small airway lesions, were only observed to be significantly different between the normal group and the PRISm group (Fig. 2C). However, EI, PI-1, and PI-15, which reflect pulmonary parenchyma destruction, were only found to be significantly different between the mild to moderate COPD group and the other groups, but MLD was significantly different among the three groups (Fig. 2B). There were no significant differences in the airway parameters or most of the vessel parameters between the PRISm group and the mild to moderate COPD group.

### Correlation between CT parameters and PFT results

The quantitative CT parameters with significant differences were selected for the correlation analysis to evaluate the pulmonary function parameters, and the results are shown in Table 2. Most of the pulmonary quantitative CT parameters had mild to moderate correlations with the pulmonary function parameters, among which the correlation between FEV1/FVC and pulmonary parenchyma attenuation parameters was the strongest, which showed a moderately significant correlation, while no significant correlation was found between the ratio of FEV1/FVC and the airway parameters.

**Table 2:** Correlation between CT parameters and pulmonary function indices

	FEV1	FEV1% pred	FVC	FEV1/FVC	VC%	DLco%	RV/TLC
EI	− 0.17 (0.02)	− 0.22 (0.002)	0.22 (0.002)	− 0.51 (< 0.001)	0.15 (0.04)	− 0.17 (0.02)	0.28 (< 0.001)
ATI	− 0.16 (0.03)	− 0.22 (0.002)	0.04 (0.58)	− 0.28 (< 0.001)	− 0.01 (0.94)	− 0.17 (0.02)	0.25 (< 0.001)
Inspiratory MLD	− 0.05 (0.51)	0.005 (0.95)	− 0.32 (< 0.001)	0.29 (< 0.001)	− 0.27 (< 0.001)	0.05 (0.48)	− 0.10 (0.18)
PI-1	0.19 (0.01)	0.25 (< 0.001)	− 0.17 (0.02)	0.51 (< 0.001)	− 0.13 (0.08)	0.20 (0.006)	− 0.28 (< 0.001)
PI-15	0.12 (0.10)	0.19 (0.009)	− 0.25 (< 0.001)	0.48 (< 0.001)	− 0.17 (0.02)	0.16 (0.03)	− 0.25 (< 0.001)
PRM^Emph^	− 0.18 (0.01)	− 0.27 (< 0.001)	0.23 (0.002)	− 0.55 (< 0.001)	0.13 (0.07)	− 0.21 (0.003)	0.30 (< 0.001)
PRM^fSAD^	− 0.20 (0.006)	− 0.25 (< 0.001)	0.01 (0.86)	− 0.35 (< 0.001)	− 0.04 (0.63)	− 0.23 (0.002)	0.28 (< 0.001)
PRM^Normal^	0.25 (< 0.001)	0.33 (< 0.001)	− 0.07 (0.30)	0.49 (< 0.001)	0.01 (0.90)	− 0.27 (< 0.001)	− 0.33 (< 0.001)
Pi10	− 0.37 (< 0.001)	− 0.33 (< 0.001)	− 0.36 (< 0.001)	− 0.11 (0.14)	− 0.32 (< 0.001)	− 0.03 (0.64)	0.23 (0.001)
Wall area	− 0.30 (< 0.001)	− 0.25 (0.001)	− 0.27 (< 0.001)	− 0.13 (0.08)	− 0.24 (0.001)	− 0.04 (0.55)	0.30 (< 0.001)
Lumen area	0.28 (< 0.001)	0.26 (< 0.001)	0.31 (< 0.001)	0.04 (0.59)	0.27 (< 0.001)	− 0.04 (0.62)	− 0.18 (0.01)
Number of vessels	0.20 (0.005)	0.16 (0.03)	0.10 (0.18)	0.22 (0.003)	0.08 (0.28)	0.18 (0.01)	− 0.13 (0.06)
Number of vessels under 5 mm^2^	0.24 (0.001)	0.21 (0.004)	0.11 (0.11)	0.25 (< 0.001)	0.11 (0.12)	0.20 (0.006)	− 0.17 (0.02)
Average diameter of vessels	− 0.26 (< 0.001)	− 0.31 (< 0.001)	− 0.15 (0.04)	− 0.20 (0.004)	− 0.26 (< 0.001)	− 0.16 (0.03)	0.23 (0.001)
Surface LAA	− 0.12 (0.11)	− 0.23 (0.001)	0.29 (< 0.001)	− 0.51 (< 0.001)	0.16 (0.03)	− 0.21 (0.003)	0.28 (< 0.001)

Data are Spearman r correlation values. Numbers in parentheses are p values

The correlations between PRM, the airway parameters, the vascular parameters and FEV1 were stronger than that of the parenchyma attenuation parameters. The correlation between VC% and the airway parameters was relatively strong compared to the other parameters, while the correlation between DLCO and PRM was mild but relatively significant.

Moreover, there was a mild but relatively significant correlation between RV/TLC and most CT parameters, including emphysema, obstruction of small airways and even some vascular parameters. This might indicate that residual volume was an important marker in the early COPD progression. Tanabe et al. [12] found that both the percentage ratio of the airway tree to lung volume and low attenuation volume had greater impacts on RV/TLC than the other CT indexes. Another study [13] suggested that the ratio of MLD in expiration to inspiration which represents small airways disease was the only CT measure to independently predict RV% and RV/TLC. In our study, it was difficult to distinguish which part played a leading role in the rise of RV, or it may be the result of a combination of multiple factors. Further research is needed in this area.

### Binary logistic regression analysis

**Fig. 3:**
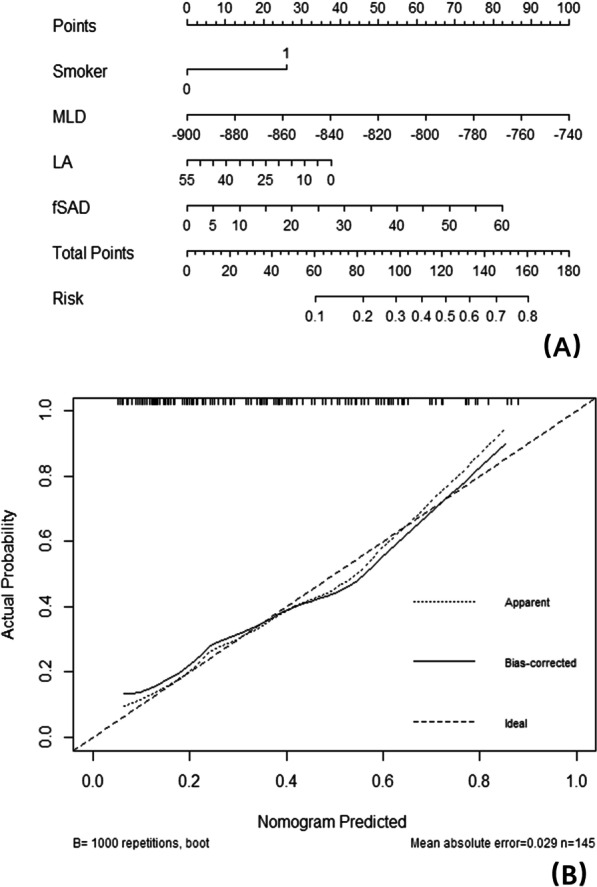
**A** nomogram of the model to predict PRISm from normal, *LA* lumen area; **B** calibration curve of the nomogram

**Fig. 4:**
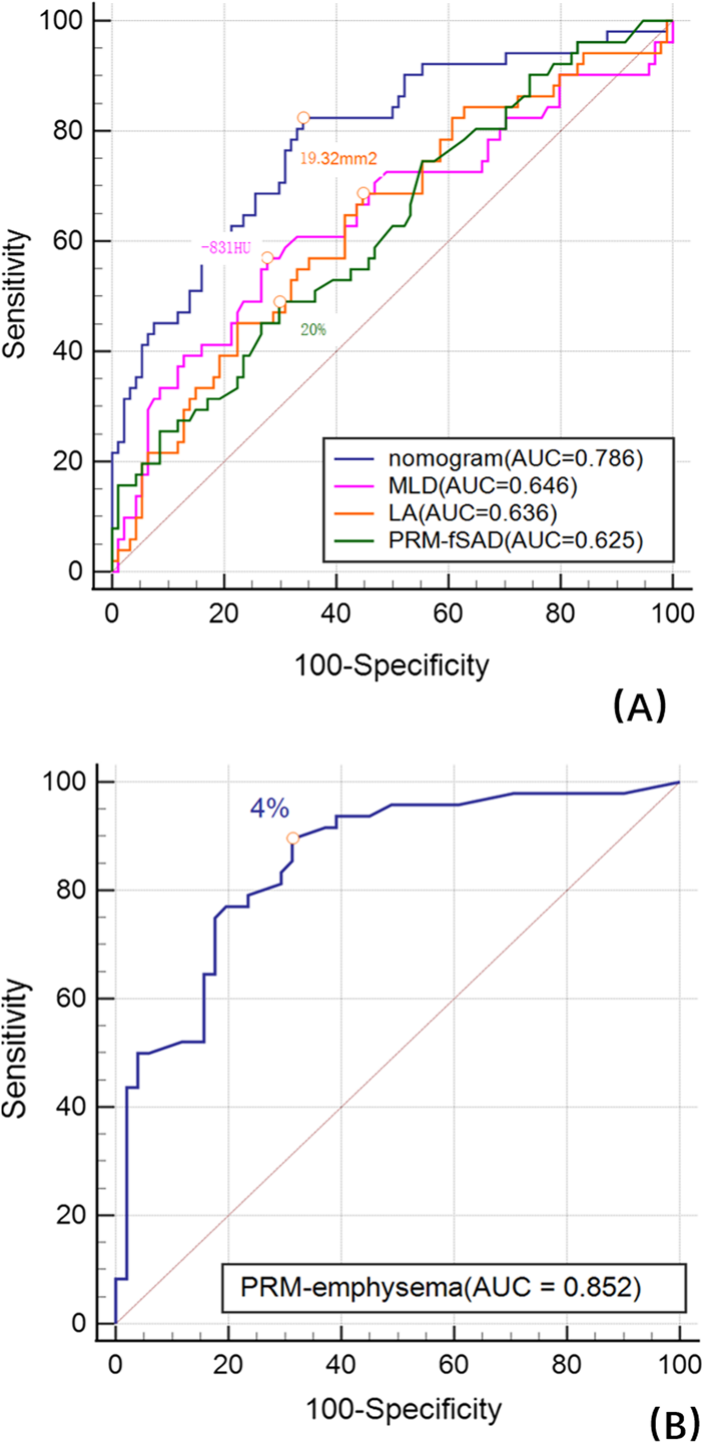
**A** ROC curve of the nomogram and parameters to predict PRISm from normal. The AUC value of the model was 0.786 (sensitivity 82.35%, specificity 65.96%). **B** ROC curve of PRM^Emph^ to predict mild to moderate COPD from PRISm. The cutoff criterion value was 4%. This indicated that when the proportion of emphysema in PRM was 4%, it was helpful to distinguish the Mild to Moderate COPD group from the PRISm Group. The sensitivity and specificity of this index were 89.58% and 68.63%, which had good significance

Table 3 shows the results of binary logistic regression analysis in the normal group and the PRISm group. The level of significance entering the model was designated as P < 0.2. A stepwise algorithm showed that the predictors within the model that distinguished the normal group from the PRISm group were smoking, MLD, PRM^fSAD^ and lumen area (Fig. 2A). The formula of the model was P = ex/(1 + ex), x = 18.134–1.072 * smoking + 0.022 * MLD + 0.05 * PRM^fSAD^ − 0.095 * Lumen area, which had a good goodness of fit (χ^2^ = 0.31, P < 0.001). The area under the curve (AUC) value of the model was 0.786 (sensitivity 82.35%, specificity 65.96%). An ROC curve analysis showed that the cutoff criterion value was − 831 HU for MLD, 20% for PRM^fSAD^, and 19.32 mm^2^ for the lumen area (Fig. 4A). The DeLong test showed that the ROC curve of the model was significantly different from that of other individual parameters (z statistic, P < 0.01). Figure 3A shows the nomogram of the model, and Fig. 3B shows the calibration curve of the nomogram, which evaluates the accuracy of the model and any potential model overfitting through a bootstrap verification of 1000 resamplings. It shows a comparison between the actual risk and predicted risk, which has good consistency. The PRISm group and the mild to moderate COPD group were also used in this method, and the predictor that distinguished between the PRISm group from mild to moderate COPD group was PRM^Emph^ (P < 0.001, OR: 1.182, 95% CI 1.097–1.274), and the AUC value was 0.852 (sensitivity 89.58%, specificity 68.63%). The ROC curve analysis showed that the cutoff criterion value was 4% (Fig. 4B).

**Table 3:** Binary logistic regression analysis for CT/clinical parameters to predict PRISm from normal

	OR (95% CI)	Coefficient (P)
Smoker	0.342 (0.122–0.957)	0.041
Inspiratory MLD	1.022 (1.008–1.036)	0.002
Lumen area	0.909 (0.827–1.000)	0.050
PRM^fSAD^	1.051 (1.004–1.100)	0.033

Stepwise algorithm selected above parameters as predictors of the model (χ^2^ = 0.31, P < 0.001), OR (95% CI) = odds ratio (95% confidence interval)

## Discussion

In this study, PRISm patients were compared with normal and mild to moderate COPD patients, and the correlations between the quantitative CT parameters and lung function parameters were analyzed. We aimed to develop a model that could predict PRISm.

One study [14] found that the risk factors for PRISm include female sex, smoking, advanced age, extreme weight and so on. A cross-sectional and follow-up study of COPDGene [8, 15] analyzed a cohort of former smokers and current smokers and found that PRISm patients had a higher BMI than normal and COPD patients, and continuous smoking was an independent predictor for a decreased quality of life in COPD patients. The population of this study differs from that of the previous study and includes both smokers and nonsmokers. No significant differences in BMI and sex were found between the three groups, but smoking was still found to be a predictive factor for distinguishing PRISm patients from the normal patients. In addition, this study found that there was no significant difference in the lung parenchymal attenuation parameters between the normal group and the PRISm group. Differences were found between the mild to moderate COPD group and the other two groups, which is consistent with the results of Wei et al. [16]. These results show that the PRISm group is more similar to the normal group in terms of parenchyma attenuation, which is quite different from the mild to moderate COPD group. Since FEV1/FVC has the strongest correlation with pulmonary parenchyma attenuation parameters, but not with airway parameters. PRISm mainly affects the airways, including small airway lesions, and has little impact on lung parenchyma. Therefore, the value of FEV1/FVC does not change significantly in PRISm, which is consistent with the definition of pulmonary function indexes of PRISm. Previous pathological studies [17, 18] have also shown that COPD is characterized by a combination of airway narrowing and frank distal airway disappearance, which silently increases airway resistance and precedes emphysema development. Therefore, it is reasonable to assume that PRISm represents an earlier stage of COPD development.

Another COPDGene study [8] found that the percentage of emphysema in PRISm patients was increased compared to the normal group, and it was considered as one of the main risk factors for PRISm. But the data was adjusted by TLC in the study and they also found that the predicted percentage of TLC decreased. When not adjusted by TLC, the percentage of emphysema did not show increase in the PRISm group. In other studies [9, 16], the percentage of emphysema in the PRISm group was not higher than that in the normal group either, and none of them were adjusted by TLC. Therefore, we think it was the decrease in TLC that masked the change in the percentage of emphysema. If we only look at the unadjusted data, the conclusions were consistent. Whether it is necessary to make the adjustments on emphysema needs to be further studied. It is worth noting that there were significant differences in the MLD of the inspiratory phase among the three groups, and it was a predictor for distinguishing the normal group from the PRISm group. The MLD of the PRISm group was higher than that of the normal group. Although our current analysis does not reveal its biological basis, previous histopathological investigations may suggest its underlying etiology. This may be related to the insufficient inflation of the lungs caused by the destruction of terminal bronchioles in the early stage of COPD [18]. The precursor to centrilobular emphysema is a gain of tissue due to inflammation and remodeling of the respiratory bronchiole and surrounding alveolar structures [19]. Additionally, smokers develop pathologic and radiological evidence of fibrosis that increases lung density [20]. Wei et al.'s research also suggests that when dyspnea occurs, the body makes compensatory adjustments to relieve symptoms. The decrease of lung capacity and the increase of mean lung density observed may be the early histological changes to overcome the increase of small airway resistance. These data show that the development in early COPD is not characterized by a simple monotonic loss of tissue, and the early changes in lung density are not necessarily linear declines.

However, changes in the small airways are difficult to measure directly on CT images. General studies [13, 21, 22] use air trapping to quantify the small airway function with biphasic CT imaging, but air trapping is caused not only by small airway diseases but also by emphysema. PRM can distinguish it well, and it is a special imaging biomarker that was proposed by Galbán et al. [23] for the accurate classification of lung density based on voxels. This technology uses dynamic image registration to identify changes in the dual-phase voxel density, thereby identifying the entire lung area as normal (PRM^Normal^), emphysema gas capture (PRM^Emph^) and non-emphysema gas capture (PRM^fSAD^). This kind of non-emphysema gas capture can reflect the functional changes of small airways, so it is called “functional small airway disease” (fSAD). Vasilescu et al [24] believe that the areas of loss, narrowing and obstruction of pulmonary terminal bronchioles can be identified based on PRM^fSAD^. Pompe et al. [25] showed that PRM^Emph^ and PRM^fSAD^ in COPD patients were higher than those in non-COPD patients and increased with an increasing GOLD stage. Bhatt et al. [26] analyzed the 5-year COPDGene follow-up data and found that the decrease in FEV1 in mild to moderate COPD was the largest, and the correlation between FEV1 and PRM^fSAD^ was the greatest. This study found that PRM also has a good differentiation for normal, PRISm and mild to moderate COPD, in which PRM^fSAD^ has a significant difference between the normal group and the PRISm group, which is a significant predictor of the model. PRM^Emph^ is an independent predictor of PRISm and mild to moderate COPD. Therefore, this study suggests that PRISm is more similar to mild to moderate COPD in terms of small airway disease, which is significantly different from that of normal patients. PRISm may represent the early stage of the development of COPD, when there is no destruction of the lung parenchyma but there are changes in the small airway, which is also consistent with the pathological results proposed by the Hogg team [18]. They believe that stenosis or disappearance of the small airway occurs before the destruction of emphysema, and disease of the small airway may be a precursor of emphysema.

In addition to the small airway, the medium airway visible in the images is also worth exploring. A bronchial tree as low as the sixth-generation airway can be generated by three-dimensional reconstruction; thus, the parameters of the airway can be analyzed. Pi10 can be calculated to measure the standardized airway wall thickness, but the research results are different [27–30], which may be due to airway measurement errors and the uncertainty of Pi10. Because the complexity of the bronchial tree is simplified to a number, some information is missing. Wei et al. [16] studied normal, PRISm and mild COPD patients with chronic bronchitis and found that compared with the normal group, the percentage of the fifth-generation bronchial wall area of the upper lobe of the left lung increased in the PRISm group. In this study, through the analysis of normal, PRISm and mild to moderate COPD populations, it was found that there was a significant difference in the airway parameters between the normal group and PRISm group, but there was no significant difference between the PRISm group and the mild to moderate COPD group. The lumen area could be used as a predictive factor to distinguish normal from PRISm. Ostridge et al. [13] found that Pi10 differs in patients with moderate and severe COPD, but it did not show an independent correlation with any pulmonary function parameters. Koo et al. [31] found that Pi10 was weakly positively correlated with FEV1/FVC in patients with GOLD 2 and 3. There was no strong correlation between the CT airway parameters and pulmonary function in moderate and severe COPD, which may be due to the dominant emphysema component masking the influence of the airway.

In addition, this study also found significant differences in most of the vessel parameters. Most studies [32–34] measure the vessel parameters by selecting three fixed cross-sectional images from the entire CT image to measure the diameter or cross-sectional area of pulmonary small vessels. In this study, the overall blood vessel data were measured by the software to explore the differences among the three groups, which was more comprehensive and improved in terms of sampling error. No significant difference was observed in the total vessel volume. This may be because the early lesions are mainly in the small blood vessels, which have little effect on the blood vessels as a whole. The other vessel parameters were measured at a distance of 6 mm away from the pleural surface. Significant differences were observed between the normal group and the PRISm group, but there was no significant difference between the PRISm group and the mild to moderate COPD group. This result was similar to our previous discovery of airway parameters, indicating that compared with the normal group, the changes in small airways and small vessels occurred in PRISm, but the attenuation of the lung parenchyma did not change significantly. The attenuation of pulmonary parenchyma was further increased in the mild to moderate COPD group compared with the PRISm group. METS et al. [35] showed that vessel parameters are related to pulmonary health indicators and the probability of airflow obstruction. Quantitative CT assessment can detect subclinical and early subtle pulmonary vascular lesions, which is consistent with the results of this study. However, the vessel parameters were not ultimately included in the prediction model, indicating that the change in vessel parameters of PRISm is not as significant as that of small airway and other parameters.

It is undeniable that CT plays an important role in the diagnosis of early COPD, because most patients have reached the middle or late stage once they have obvious symptoms. Pulmonary function test has its limitations for extensive screening of early and mild pulmonary dysfunction. If we can predict and take action as early as possible through routine lung cancer screening CT, it will greatly improve the process of COPD. Therefore, CT is particularly important in the early diagnosis of COPD. The development of airflow restriction in COPD patients includes the progressive destruction and loss of terminal and transitional bronchioles before a decline in pulmonary function. In particular, CT appears to be inherently more sensitive than PFT parameters to early stages of COPD [6], suggesting that CT can provide more information on the status of a patient compared to PFT parameters.

It is true that ionizing radiation is a problem with CT scans. A comprehensive assessment of COPD requires dual-phase CT scanning including deep inspiration and expiration, which almost double the radiation dose. Therefore, it is difficult to be widely used in daily diagnosis and treatment, which limits its clinical application. However, it is well known that small airway disease shows outstanding importance in the early stages of COPD. If we want to have the opportunity to change the outcome of COPD, future studies need to be conducted in the cohort of patients with early COPD and small airway diseases will be a signally promising direction. So dual-phase CT scanning is inevitable. Studies should focus on radiation dose reduction techniques for CT and how to predict parametric response mapping from the single inspiratory phase.

This study has some limitations. First, the number of patients was relatively small, and the clinical data were limited. The small sample size may reduce the confidence level of the study, increase the margin of error and we may have to settle for less conclusive results. Second, although these patients have undergone respiratory training before CT scanning, the degrees of inhalation and exhalation were still different in each patient, and the small differences may affect the measurement of some quantitative CT parameters. In addition, we did not include severe and extremely severe COPD patients in the study, considering that the number of patients was small and these groups were largely different from the PRISm group that we focused on. This may have an impact on the results of this study. Finally, this is a single-center study, which lacks external verification data to evaluate the robustness and practical clinical application value of the model. In the future, more suitable methods will be explored to improve the accuracy of prediction through multicenter, standardized studies with larger sample sizes.

## Conclusions

In conclusion, we confirmed that there were significant differences in quantitative CT parameters between the PRISm group, normal group and mild to moderate COPD group, and most of them had good correlations with pulmonary function parameters. The nomogram composed of smoking, MLD, PRM^fSAD^ and lumen area could distinguish the PRISm group from the normal group well, while PRM^Emph^ was an independent predictor between the PRISm group and the mild to moderate COPD group. This study has clinical significance because we focused on the imaging characteristics of PRISm, which is a special group of patients. This study will help to improve the clinical value of quantitative CT and early intervention for COPD.

## Data Availability

The datasets used and/or analysed during the current study are available from the corresponding author on reasonable request.

## Abbreviations

PRISm: Preserved ratio impaired spirometry
COPD: Chronic obstructive pulmonary disease
HRCT: High-resolution computed tomography
EI: Emphysema index
MLD: Mean lung density
fSAD: Functional small airways disease
FEV1: Forced expiratory volume in one second
FEV1%pred: Percentage predicted forced expiratory volume in one second
FVC: Forced vital capacity
FEV1/FVC: Ratio of forced expiratory volume in one second to forced vital capacity
VC: Vital capacity
ATI: Air trapping index
PRM: Parametric response mapping
LAA: Low attenuation area
ANOVA: One-way analysis of variance
