# Correction: Subtyping preserved ratio impaired spirometry (PRISm) by using quantitative HRCT imaging characteristics

**DOI:** 10.1186/s12931-023-02325-5

**Published:** 2023-01-25

**Authors:** Jinjuan Lu, Haiyan Ge, Lin Qi, Shaojie Zhang, Yuling Yang, Xuemei Huang, Ming Li

**Affiliations:** 1 Department of Radiology, Huadong Hospital Affiliated to Fudan University, 221 West Yanan Road, Jingan District, Shanghai, 200040 China; 2 Department of Respiratory Medicine, Huadong Hospital Affiliated to Fudan University, Shanghai, China

**Correction: Respiratory Research (2022) 23:309** https://doi.org/10.1186/s12931-022-02113-7

Following publication of the original article [1], the authors would like to update the funding section.

It has been updated in this correction.

The updated Funding section should read as below:

**Funding**

This study was supported by the National Natural Science Foundation of China (61976238); Shanghai Municipal Science and Technology Commission (21Y11910500); Shanghai Municipal Health Commission (20204Y0299); Medical Imaging Key Program of Wise Information Technology of 120, Health Commission of Shanghai 2018ZHYL0103 (ML), “Future Star” of famous doctors’ training plan of Fudan University. This study was also supported by the Shanghai Municipal Science and Technology Commission (20Y11902900).

The original article has been corrected.

## References

[1] Lu J, Ge H, Qi L, Zhang S, Yang Y, Huang X, Li M (2022). Subtyping preserved ratio impaired spirometry (PRISm) by using quantitative HRCT imaging characteristics. Respir Res.

